# Tele-Electrocardiography and Mortality: Clinical Outcomes in Digital Electrocardiography Cohort–Data from Belo Horizonte, Brazil (CODE-BH)

**DOI:** 10.5334/gh.1554

**Published:** 2026-05-22

**Authors:** Gabriela Miana de Mattos Paixão, Carla Paula Moreira Soares, Paulo Gomes Rodrigues, Peter W. Macfarlane, Antonio Luiz P. Ribeiro

**Affiliations:** 1 Telehealth Center Hospital das Clínicas of the Universidade Federal de Minas Gerais (UFMG), Belo Horizonte, MG, Brazil; 2 Faculty of Medicine at the UFMG, Belo Horizonte, MG, Brazil; 3 School of Health and Wellbeing, College of Medical, Veterinary and Life Sciences, University of Glasgow, Glasgow, Scotland, UK

**Keywords:** electrocardiogram, electronic cohort, cardiovascular hospitalization, mortality

## Abstract

Cardiovascular diseases are the leading cause of mortality in Brazil and around the world. The electrocardiogram (ECG) has a vital diagnostic and prognostic role in cardiovascular diseases. The CODE-BH cohort was established by linking ECG data (2006 to 2018) to hospitalization and mortality data from the public health database of Belo Horizonte, Minas Gerais, Brazil. It comprises 474,764 ECGs from 337,021 patients (mean age 54.42 years; 38.2% male), mostly from primary care centers. Clinical data were collected using a standardized questionnaire. ECG exams, hospitalization, and mortality information system data were linked using probabilistic linkage methods. The mortality rate was 3.4% (n = 11,518) with a mean follow-up of 3.3 years. Mortality was associated with older age, male sex, presence of any comorbidities, and major ECG abnormalities (p < 0.001). The presence of any ECG abnormality by Minnesota Code was associated with a higher risk of mortality (95% CI HR 1.45 (1.36–1.54) for minor abnormalities; HR 2.30 (2.16–2.44) for major abnormalities; p < 0.001), and cardiovascular hospitalization (95% CI HR 1.39 (1.28–1.50) for minor abnormalities; HR 3.12 (2.89–3.37) for major abnormalities; p < 0.001). This cohort brings novel data for epidemiological research. It encompasses a large amount of data from ECGs linked to clinical outcomes from a Latin American population.

## Graphic Abstract

**Figure:**
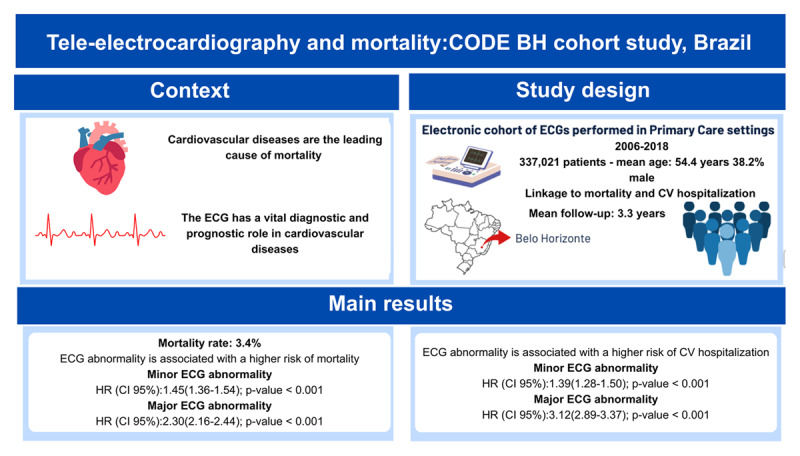

## Introduction

Cardiovascular diseases are the leading cause of mortality in Brazil and the world (1). The electrocardiogram (ECG) is a low-cost, easily accessible, and non-invasive exam that is part of both the initial assessment of the patient in the investigation of heart disease, as well as the clinical follow-up of patients with an established cardiac condition. The ECG has a vital diagnostic and prognostic role in cardiovascular diseases.

The digital ECG has introduced new possibilities for epidemiological and clinical research. Many simple, widely available exams with clinical importance can now be linked to electronic health records, providing reliable information in short, middle, and long-term follow-ups within population-based studies. While several electronic cohort studies in electrocardiography have been developed previously—from prevalence studies to identifying prognostic factors (2,3,4,5)—the emergence of artificial intelligence (AI) has enabled new ECG risk markers to be described (6), expanding the diagnostic and prognostic potential of the ECG (7,8).

In lower-middle-income countries such as Brazil, few extensive population studies aim to assess cardiovascular prognosis. Despite some solid and well-developed telecardiology services (9), there are several limitations to obtaining electronic population cohorts, such as the incomplete implementation of electronic medical records in primary and secondary care centers, the lack of integration of the medical record network with information systems, and the lack of interface between outpatient, hospital, and mortality information systems. As the Universidade Federal de Minas Gerais (UFMG) operates a telehealth service that provides teleECG to small counties (10), we built an electronic cohort with linked data from an extensive database of digital ECGs with hospitalizations and deaths obtained from public health records. The present report describes the development of this electronic cohort, provides preliminary results regarding mortality and cardiovascular hospitalization, and discusses its challenges and potential applications.

## Methods

### Telehealth Network of Minas Gerais

The Telehealth Network of Minas Gerais (TNMG) (9) is a collaborative telehealth service of seven public universities in Minas Gerais, Brazil. Minas Gerais is the Brazilian state with the highest number of municipalities (853) and the second-highest population. It can be considered representative of the country, as age distribution, social inequality, and percentage of urbanization are similar to the overall national pattern. TNMG was implemented in partnership with the government in 2005 to improve health assistance to small counties. Since 2017, the TNMG has expanded its services to national boundaries. The TNMG is one of the most important telehealth networks in Latin America and performs different modalities of telehealth activities across specialties, including assistance services, education, research, and innovation development.

The TNMG telecardiology service covers more than 1,400 municipalities from 14 Brazilian states and has analysed more than ten million ECGs since its inception. Most of these municipalities (952, 72.1%) have up to 20,000 inhabitants and only one registered ECG device. Across the serviced areas, 85% of ECG machines are in primary healthcare units, while 15% are in small hospitals and emergency care units. Since 2018, the tele-ECG service has operated 24 hours a day, seven days a week, with at least two cardiologists available each shift to make the ECG report and perform synchronous teleconsultations. Elective ECG reports were issued and sent to the requesting health unit in a median of 41’30” (P25 = 6’44”, P75 = 1h32’24”) and emergency reports in a median of 47” (P25 = 22”, P75 = 1’55”). In addition to the assistance service, extensive education material in cardiology—including ECG interpretation and management of cardiovascular diseases and their risk factors—is available through the TNMG’s website and main social media channel (10).

### The CODE (Clinical Outcomes in Digital Electrocardiography) cohort

The CODE study started in 2017 as an initiative to consolidate and organize the database of digital ECG exams of the TNMG, linking it to the public databases of the Mortality and Hospitalization Information Systems (11). The ECGs obtained by the TNMG from 2010 to 2017 were organized in a structured database. We utilized an artificial intelligence algorithm based on natural language processing to automatically recognize diagnostic categories within free-text ECG reports generated by cardiologists. The algorithm was developed to identify and classify key phrases that correspond to electrocardiographic diagnoses, thereby transforming unstructured narrative reports into structured data suitable for clinical and epidemiological analysis. The ECG database was linked to the Brazil Mortality Information System using probabilistic linkage methods. From 2,470,424 ECGs, 1,773,689 unique patients were identified. A subset of this dataset (15%) is publicly available online (12). Studies from the CODE cohort have been published regarding the predictive value of traditional ECG abnormalities (13,14,15,16) and assessing the diagnostic and prognostic aspects using artificial intelligence (6,17). Several research groups have collaborated with this dataset alongside the TNMG team to develop AI tools (18,19,20,21,22).

### The CODE-BH cohort

The CODE-BH cohort is an extension of the CODE cohort, but includes only patients from the capital of Minas Gerais, Belo Horizonte. Since hospitalization data from the national public records are not openly available, we evaluated data only from a subset of patients.

**Figure 1:**
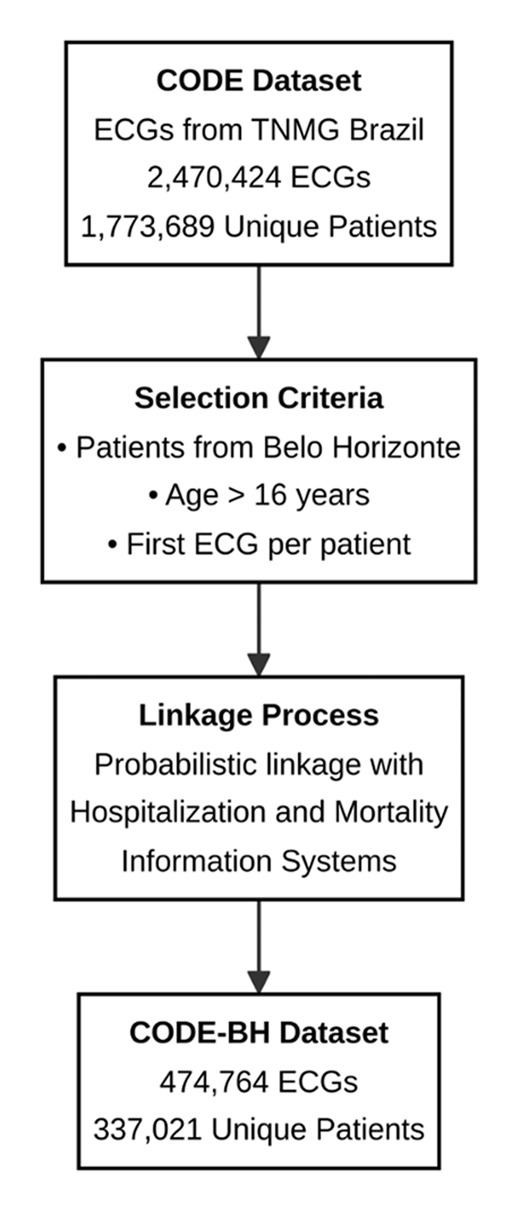
Flowchart of the data processing and patient selection procedures for the CODE and CODE-BH cohorts.

All ECGs performed by the TNMG in Belo Horizonte on patients over 16 years old from 2006 to 2018 were assessed. For patients with more than one ECG, only the first exam was included in the analysis (Figure 1). The majority of patients were from primary care centers. The electronic cohort was obtained by linking data from the ECG exams (name, sex, date of birth, city of residence) and from the hospitalization and Mortality Information System, using standard probabilistic linkage methods (FRIL: Fine-grained record linkage software, v.2.1.5, Atlanta, GA). The data was anonymized for storage after the ECG database linkage with hospitalization and mortality records. The data is stored in a PostgreSQL database, with the most important fields for analysis being patient clinical data, ECG tracing, lead signals, text reports, diagnostic statements, Minnesota codes, and data from the hospitalization document along with death certificate (where appropriate).

The study was approved by the Federal University of Minas Gerais Research Ethics Committee (CAAE: 68496317.7.3001.5140), which waived the informed consent form.

### Acquisition, processing, and analysis of the ECG data

All 12-lead ECGs analyzed were obtained by the TNMG using a Web application built on the Java programming language. ECGs were recorded by the local primary care professional using an electrocardiograph model TEB ECGPC, manufactured by Tecnologia Eletrônica Brasileira (São Paulo, Brazil), or a model ErgoPC 13 by Macromedia Biotecnologia (Brasilia, Brazil). Tracings obtained by these ECG machines were sent to central servers via the internet, using the web application developed in-house. The duration of the ECG recordings was between seven and ten seconds, sampled at frequencies ranging from 300 to 1000 samples per second. A standardized questionnaire was completed by a technical nurse to collect self-reported information on age, sex, comorbidities, current symptoms, and medications. Standardized training for clinical questionnaire administration was implemented at the time of service deployment and replicated across all teams to promote uniform data collection procedures, though data quality may vary across different collection sites and time periods. Clinical conditions included self-reported smoking, hypertension, diabetes, dyslipidemia, Chagas disease, previous myocardial infarction, and chronic obstructive pulmonary disease.

The clinical information, ECG tracings, and reports were stored and then analyzed using the Glasgow 12-lead ECG program (23) (release 28.4.1, issued on June 16th, 2009), providing a complete interpretation together with Minnesota codes (23,24) and ECG measurements. This paper uses only the Minnesota Code (25) to classify the ECG findings into normal, minor, and major. Minor abnormalities were: minor isolated Q/QS waves (MC 1-3), minor ST/T abnormalities (MC 4-3, 4-4, 5-3, 5-4), high R wave (left ventricular) (MC 3-1, 3-3, 3-4), high R wave (right ventricular) (MC 3-2), ST segment elevation (MC 9-2), incomplete RBBB (MC 7-3), incomplete LBBB (MC 7-6, 7-7), minor QT prolongation (QT index ≥ 112%), short PR interval (MC 6-5), long PR interval (MC 6-3), left axis deviation (MC 2-1), right axis deviation (MC 2-2), premature supraventricular beats (MC 8-1-1), premature ventricular beats (MC 8-1-2), premature combined beats (MC 8-1-3, 8-1-5), wandering atrial pacemaker (MC 8-1-4), sinus tachycardia (MC 8-7), sinus bradycardia (MC 8-8), supraventricular rhythm persistent (MC 8-4-1), low voltage QRS (MC 9-1), high amplitude P wave (MC 9-3), left atrial enlargement (MC 9-6), and fragmented QRS (MC 7-1-0).

The following were considered to be major abnormalities: major Q waves (old myocardial infarctions, MC 1-1, 1-2), minor Q waves plus ST-T abnormalities (possible old myocardial infarction MC 1-3 plus MC 4-1 or 4-2 or 5-1 or 5-2), major isolated ST-T abnormalities (MC 4-1 or 4-2 or 5-1 or 5-2), left ventricular hypertrophy plus ST-T abnormalities (MC 3-1 plus MC 4-1 or 4-2 or 5-1 or 5-2), intraventricular conduction abnormalities (complete/intermittent right and left bundle branch block, non-specific intraventricular block, MC 7-1 or 7-2 or 7-4), right bundle branch block plus left anterior divisional block (MC 7-8), major QT prolongation index (QTi ≥ 116%), atrial fibrillation/flutter (MC 8-3), supraventricular tachycardia (MC 8-4-2), atrioventricular (AV) conduction abnormalities (second and third degree AV block (MC 6-2), artificial pacemaker (MC 6-8), ventricular pre-excitation such as Wolff Parkinson White syndrome (MC 6-4).

R program (version 4.1, Vienna, Austria) was used for statistical analysis. Categorical data were reported as counts and percentages; continuous variables were reported as mean and SD. The Kruskal-Wallis test was used to compare three groups (normal ECG, minor abnormalities, or major abnormalities) and provided the global value. Comparisons between two groups (normal vs. minor, normal vs. major, minor vs. major) were performed using the Bonferroni test. Cox regression was used to assess the relation between ECG abnormality and mortality or cardiovascular hospitalization, adjusting for age, sex, and comorbidities. A confidence interval of 95% was used. A two-tailed P-value of 0.05 was considered statistically significant. In addition, survival curves were computed using Kaplan-Meier estimates to examine the association between ECG and all-cause mortality.

## Results from the CODE-BH cohort

From 2006 to 2018, 474,764 electrocardiograms (ECGs) were acquired in Belo Horizonte. This dataset comprised 337,021 distinct patients, with a mean age of 54.42 years; 128,398 (38.2%) were male. The predominant clinical condition among the identified individuals was hypertension. The ECG findings were further categorized into three groups: normal (n = 124,816; 35.3%), minor abnormalities (n = 132,266; 39.3%), and major abnormalities (n = 79,939; 25.4%), according to the Minnesota code. Table 1 summarizes all clinical data according to the presence of major ECG abnormalities and presents comparisons between each group, considering age, sex, and comorbidities. Patients with major abnormalities were predominantly older males and had more clinical conditions when compared to patients with minor abnormalities or normal ECG. In the latter, only smoking did not have a significant difference. Patients with minor ECG abnormality were also predominantly older males and had a higher prevalence of hypertension, diabetes, dyslipidemia, and previous myocardial infarction when compared to patients with normal ECG.

**Table 1:** Clinical characteristics of CODE-BH patients, according to the Minnesota code.


VARIABLE n;(%)	NORMAL ECG n = 124,816	MINOR ECG ABNORMALITY n = 132,266	MAJOR ECG ABNORMALITY n = 79,939	GLOBAL p-VALUE	NORMAL VS MINOR p-VALUE	NORMAL VS MAJOR p-VALUE	MINOR VS MAJOR p-VALUE

Age (mean, SD)	48.7 (16.4)	52 (18.1)	58.2 (18.3)	<0.001	<0.001	<0.001	<0.001

Gender				<0.001	<0.001	<0.001	<0.001

Female	84,428 (67.6)	76,629 (57.9)	47,566 (59.5)				

Male	40,388 (32.4)	55,637 (42.1)	32,373 (40.5)				

Hypertension	32,679 (45.0)	40,264 (54.1)	28,727 (63.7)	<0.001	<0.001	<0.001	<0.001

Diabetes	13,867 (11.1)	17,667 (13.4)	13,200 (16.5)	<0.001	<0.001	<0.001	<0.001

Smoking	9,015 (7.2)	9,777 (7.4)	5,638 (7.1)	0.01	0.10	0.15	0.004

Dyslipidemia	8,286 (6.6)	10,039 (7.6)	7,554 (9.4)	<0.001	<0.001	<0.001	<0.001

MI	1,238 (1.0)	1,767 (1.3)	2,411 (3.0)	<0.001	<0.001	<0.001	<0.001

Chagas disease	508 (0.4)	589 (0.4)	817 (1.0)	<0.001	0.14	<0.001	<0.001

COPD	1,276 (1.0)	1,381 (1.0)	1,009 (1.3)	<0.001	0.60	<0.001	<0.001


Data are presented as mean(SD) or number (%). COPD: Chronic obstructive pulmonary disease, MI: Myocardial infarction.

Most major abnormalities were related to myocardial ischemia (n = 79,000; 71.7%), represented by major ST segment changes and q waves. Intraventricular conduction blocks were present in 1,555 patients (14.1%), followed by arrhythmias (n = 7,302; 6.7%), mainly atrial fibrillation/flutter (n = 7,089; 6.5%). Left ventricular hypertrophy with ST-segment abnormalities was present in 5,568 patients (5.1%), and 1,872 patients (1.7%) had pacemakers. High-degree atrioventricular blocks were reported in 413 patients (0.3%), as described in Table 2.

**Table 2:** Prevalence of ECG major abnormality by gender.


MAJOR ABNORMALITY	N (%)	FEMALE (%)	MALE (%)

**Ischemic**	**79,000 (71.7)**	**47,856 (60.6)**	**31,144 (39.4)**

ST segment	39,075 (35.5)	23,766 (60.8)	15,309 (39.2)

q wave	36,270 (32.9)	22,065 (60.8)	14,205 (39.2)

q wave + ST segment	3,655 (3.3)	2,025 (55.4)	1,630 (44.6)

**IV conduction**	**15,555 (14.1)**	**8,438 (54.2)**	**7,117 (45.8)**

RBBB	7,599 (6.9)	3,779 (49.7))	3,820 (50.3)

LBBB	4,785 (4.3)	3,082 (64.4)	1,703 (35.6)

Nonspecific BBB	3,171 (2.9)	1,577 (49.7)	1,594 (50.3)

**Arrhythmia**	**7,302 (6.7)**	**3,841 (52.6)**	**3,463 (47.4)**

AF/Flutter	7,089 (6.5)	3,720 (52.5)	3,369 (47.5)

SVT	13 (0.0)	7 (53.8)	6 (46.2)

**LVH with ST-segment abnormalities**	**5,568 (5.1)**	**2,993 (53.8)**	**2,575 (46.2)**

**Miscellaneous**	**2,178 (2.0)**	**1,217 (55.9)**	**961 (44.1)**

Pacemaker	1,872 (1.7)	1,050 (56.1)	822 (43.9)

WPW	306 (0.3)	167 (54.6)	139 (45.4)

**AV conduction**	**413 (0.3)**	**252 (61.0)**	**161 (39.0)**

Second and third-degree AVB	413 (0.3)	252 (61.0)	161 (39.0)


AF: atrial fibrillation, AV: atrioventricular, AVB: atrioventricular block, BBB: bundle branch block, IV: intraventricular, LBBB: left bundle branch block, LVH: left ventricular hypertrophy, RBBB: right bundle branch block, SVT: supraventricular tachycardia, WPW: Wolf-Parkinson-White.

The overall mortality rate was 3.4% (n = 11,518) in a mean follow-up of 3.3 years, up to 10 years. Mortality was associated with older age, male sex, presence of any clinical conditions, and major ECG abnormalities (Table 3).

**Table 3:** Clinical and ECG characteristics of CODE-BH patients by mortality (a) and cardiovascular hospitalization (b).


A). MORTALITY			

VARIABLE n (%)	NON MORTALITY	MORTALITY	P-VALUE

**Age (mean, SD)**	51.7 (17.8)	67.8 (14.0)	<0.001

**Sex**			<0.001

Female	202,689 (62.3)	5,933 (51.5)	

Male	122,814 (37.7)	5,585 (48.5)	

**Hypertension**	96,203 (52.3)	5,467 (66.7)	<0.001

**Diabetes**	42,402 (13.0)	2,332 (20.2)	<0.001

**Smoking**	23,316 (7.2)	1,114 (9.7)	<0.001

**Dyslipidemia**	24,799 (7.6)	1,080 (9.4)	<0.001

**MI**	4,615 (1.4)	346 (3.0)	<0.001

**Chagas disease**	1,319 (0.4)	93 (0.8)	<0.001

**COPD**	3,014 (0.9)	194 (1.7)	<0.001

**ECG**			<0.001

Normal	122,727 (37.7)	2,089 (18.1)	

Minor	128,155 (39.4)	4,111 (35.7)	

Major	74,621 (22.9)	5,318 (46.2)	

**B). CARDIOVASCULAR HOSPITALIZATION**			

**VARIABLE n (%)**	**NON HOSPITALIZATION**	**HOSPITALIZATION**	**P-VALUE**

**Age (mean, SD)**	52.0 (17.9)	64.0 (13.1)	<0.001

**Sex**			<0.001

Female	204,534 (62.3)	4,088 (47.9)	

Male	123,948 (37.7)	4,451 (52.1)	

**Hypertension**	97,617 (52.3)	4,053 (74.6)	<0.001

**Diabetes**	42,791 (13.0)	1,943 (22.8)	<0.001

**Smoking**	23,615 (7.2)	815 (9.5)	<0.001

**Dyslipidemia**	24,520 (7.5)	1,359 (15.9)	<0.001

**MI**	4,210 (1.3)	751 (8.8)	<0.001

**Chagas disease**	1,335 (0.4)	77 (0.9)	<0.001

**COPD**	3,111 (0.9)	97 (1.1)	0.08

**ECG**			<0.001

Normal	123,398 (37.6)	1,418 (16.6)	

Minor	129,631 (39.5)	2,635 (30.9)	

Major	75,453 (23.0)	4,486 (52.5)	


COPD: Chronic obstructive pulmonary disease, MI: Myocardial infarction.

Cardiovascular hospitalization was associated with older age, male sex, major ECG abnormalities, and the presence of any clinical condition except COPD (Table 3). During hospitalization, 1,791 (1.1%) patients died. After their final discharge from the hospital, 13,770 (8.1%) patients progressed to death in a mean time of 971 days. Stroke was the most prevalent cause (n = 5,413; 29.9%) of cardiovascular hospitalization, followed by heart failure (n = 5,185; 28.7%), coronary angioplasty (n = 3,345; 18.5%), myocardial infarction (n = 2,206; 12.2%), coronary artery bypass grafting (n = 1,024; 5.7%), pacemaker implantation (n = 644; 3.6%) electrophysiology study and ablation (n = 210; 1.2%), implantable cardioverter-defibrillator (n = 27; 0.1%) and cardiac resynchronization therapy implantation (n = 23; 0.1%).

**Figure 2:**
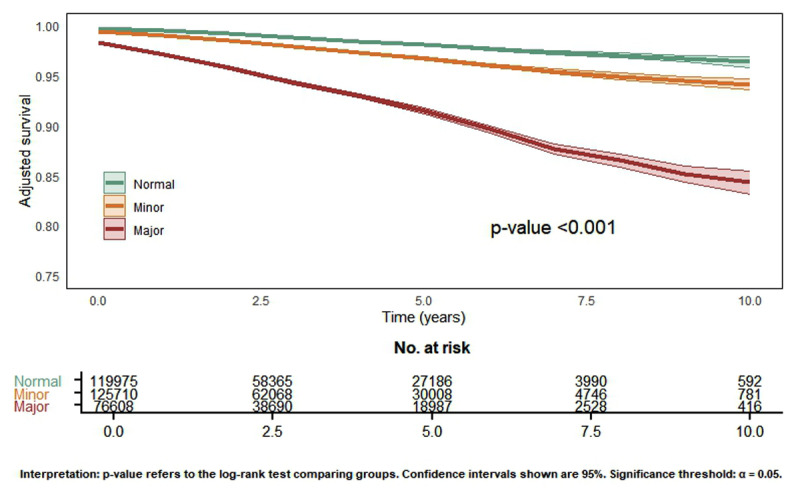
Kaplan-Meier survival curves, according to the ECG abnormality.

The presence of any ECG abnormality by the Minnesota Code was associated with a higher risk of overall mortality and cardiovascular hospitalization after adjustment for age, gender, and clinical conditions when compared to patients with a normal ECG (Table 4). In patients with major ECG abnormalities, the risk of mortality and cardiovascular hospitalization was higher than in those with minor abnormalities (Figure 2).

**Table 4:** Risk for mortality and cardiovascular hospitalization in CODE-BH cohort, according to ECG abnormality.


		MORTALITY		HOSPITALIZATION	
	
MODEL	ECG ABNORMALITY	HR (95% CI)	p-VALUE	HR (95% CI)	p-VALUE

Unadjusted	Minor	1.87 (1.77–1.97)	<0.001	1.76 (1.65–1.88)	<0.001

	Major	4.01 (3.81- 4.22)	<0.001	4.90 (4.62- 5.20)	<0.001

Adjusted by age and gender	Minor	1.43 (1.35–1.50)	<0.001	1.47 (1.37–1.56)	<0.001

	Major	2.24 (2.13–2.36)	<0.001	3.36 (3.16–3.58)	<0.001

Adjusted by comorbidities*	Minor	1.45 (1.36–1.54)	<0.001	1.39 (1.28–1.50)	<0.001

	Major	2.30 (2.16–2.44)	<0.001	3.12 (2.89–3.37)	<0.001


*Hypertension, diabetes, smoking, dyslipidemia, myocardial infarction, Chagas disease, and chronic obstructive pulmonary disease.

## Discussion

The CODE-BH cohort is one of the largest datasets of ECG data and clinical outcomes from a Latin American population. This study demonstrated that the presence of any ECG abnormality, as defined by the Minnesota Code, was significantly associated with a higher risk of overall mortality and cardiovascular hospitalization. Specifically, patients with major ECG abnormalities had a higher risk of these adverse outcomes than those with minor abnormalities or normal ECGs. These findings reaffirm the prognostic value of the ECG in a large, real-world primary care population from a middle-income country.

Our results align with previous large-scale epidemiological studies conducted in different settings, which have consistently shown that ECG abnormalities are independent predictors of cardiovascular events and mortality (26,27,28). For instance, even in a cohort of Korean patients with low cardiovascular risk, the presence of major ECG abnormalities was associated with higher overall and cardiovascular mortality. The risk of overall mortality was lower than in our study, likely due to the lower-risk profile of that population (26). Minor and major ECG abnormalities have been associated with increased cardiovascular risk in Asian and North American populations (27,28,29), with evidence of a dose-response relationship in patients with multiple concurrent ECG abnormalities (28,30). Nevertheless, our study extends these findings to a Latin American population, which has distinct clinical, racial, and social characteristics compared with those in high-income countries, which account for the majority of scientific publications.

An interesting finding was that the risk of mortality and cardiovascular hospitalization adjusted only for age and sex yielded results very similar to those of models adjusted for comorbidities. This suggests that the ECG captures a significant portion of the prognostic information related to the patient’s underlying cardiovascular health and cumulative risk factor burden. The structural and electrical changes reflected in the ECG may act as a final common pathway for various comorbidities. In resource-constrained settings where comprehensive clinical data or laboratory tests may be unavailable, the ECG, combined with basic demographic data (age and sex), can serve as a powerful tool for risk stratification (31).

Our study has several limitations that should be acknowledged. First, the observational and retrospective nature of the cohort limits our ability to establish causality between ECG abnormalities and the observed outcomes. Second, although we adjusted for several important comorbidities, residual confounding from unmeasured variables such as lifestyle factors, medication adherence, or socioeconomic status remains possible. Third, the clinical data were obtained from electronic health records and administrative databases, which may be subject to coding errors or underreporting of certain conditions. Finally, while our cohort is highly representative of the primary care population in our region, the findings may not be entirely generalizable to specialized tertiary care settings or to populations with different ethnic and demographic profiles.

Despite these limitations, this is a unique dataset with a large volume of data, enabling the identification of subclinical abnormalities and early markers of cardiac risk that might otherwise go undetected. New ECG indices can also be generated using machine learning methods to identify individuals at higher risk of death or developing new ECG abnormalities, such as incident atrial fibrillation and bundle branch block (18). This approach facilitates the development of predictive models that stratify individuals by cardiovascular risk with greater precision, supporting personalized prevention strategies. Furthermore, the continuous integration of clinical outcomes with ECG findings enhances our ability to evaluate prognostic patterns, ultimately informing public health initiatives and clinical decision-making in cardiovascular care.

With the ongoing advancement of artificial intelligence in the ECG field, the CODE cohort has the potential to develop new deep learning techniques for ECG classification and to evaluate emerging AI features, such as ECG age, that can serve as auxiliary tools in cardiac risk stratification. When implementing AI algorithms, external validation across different scenarios is mandatory. The CODE-BH presents an opportunity to establish international collaborations to develop and validate the external performance of other AI-ECG algorithms, thereby democratizing AI globally. Moreover, further studies in the AI field include methods developed to recognize established markers of cardiovascular risk—such as left ventricular systolic dysfunction—enabling the identification of subjects whose ECGs suggest they may benefit from an echocardiogram. If proven effective, this approach could be implemented in telehealth networks to improve resource utilization within the healthcare system, particularly in resource-constrained regions.

## Conclusions

Electrocardiography has an established role in the care of patients with documented or suspected cardiovascular disease. The presence of major and minor ECG abnormalities is an independent risk marker of increased mortality and cardiovascular hospitalizations in this large primary care population. The availability of a large electronic health record cohort alongside the continued development of artificial intelligence techniques can further advance our understanding of electrocardiography’s role in clinical practice and open new applications for its use. Thus, the CODE-BH dataset can be helpful for further developments in digital electrocardiography, clinical cardiology, and cardiovascular epidemiology.

## Data Availability

The data underlying this study are available in the published article, and the raw data supporting the findings are available from the corresponding author upon reasonable request.
